# Eleven immune-gene pairs signature associated with TP53 predicting the overall survival of gastric cancer: a retrospective analysis of large sample and multicenter from public database

**DOI:** 10.1186/s12967-021-02846-x

**Published:** 2021-04-29

**Authors:** Junyu Huo, Liqun Wu, Yunjin Zang

**Affiliations:** 1 Liver Disease Center, The Affiliated Hospital of Qingdao University, No. 59 Haier Road, Qingdao, 266003 China; 2 Qingdao University, No. 308 Ningxia Road, Qingdao, 266071 China

**Keywords:** Gastric cancer, Immune, Gene pairs, Prognostic, Signature

## Abstract

**Background:**

Growing attention have been paid to the relationship between TP53 and tumor immunophenotype, but there are still lacking enough search on the field of gastric cancer (GC).

**Materials and methods:**

We identified differential expressed immune-related genes (DEIRGs) between the TP53-altered GC samples (n = 183) and without TP53-altered GC samples (n = 192) in The Cancer Genome Atlas and paired them. In the TCGA cohort (n = 350), a risk score was determined through univariate and multivariate cox regression and Lasso regression analysis. Patients were divided into two groups, high-risk and low-risk, based on the median risk score. Four independent cohorts (GSE84437,n = 431; GSE62254, n = 300; GSE15459, n = 191; GSE26901, n = 100) from the Gene Expression Omnibus (GEO) database were used to validate the reliability and universal applicability of the model.

**Results:**

The signature contained 11 gene pairs showed good performance in predicting progression-free survival (PFS), disease-free survival (DFS), disease special survival (DSS), and the overall survival (OS) for GC patients in the TCGA cohort. The subgroup analysis showed that the signature was suitable for GC patients with different characteristics. The signature could capable of distinguish GC patients with good prognosis and poor prognosis in all four independent external validation cohorts. The high- and low-risk groups differed significantly in the proportion of several immune cell infiltration, especially for the T cells memory resting, T cells memory activated and follicular helper, and Macrophage M0, which was also related to the prognosis of GC patients.

**Conclusion:**

The present work proposed an innovative system for evaluating the prognosis of gastric cancer. Considering its stability and general applicability, which may become a widely used tool in clinical practice.

**Supplementary Information:**

The online version contains supplementary material available at 10.1186/s12967-021-02846-x.

## Background

Gastric cancer (GC) is a typical malignant tumor in clinical practice. Its incidence rate and mortality rate were the second highest in the world [1]. Surgery combined with radiotherapy and chemotherapy is the main method for the treatment of GC. However, due to the occult early symptoms of GC, most patients were in the advanced stage when diagnosed, and less than 20% could survive 5 years [2]. With the wide promotion of precision medicine, the research on gene molecular targeted precise therapy has become a hot topic in the field of cancer.

The high mutation rate of TP53 in tumors makes it a very attractive potential therapeutic target [3]. In the cell cycle, normal p53 is activated during DNA damage or hypoxia, which stagnates the cell cycle at G1/S point and carried out DNA repair. If the repair fails, downstream genes were activated to induce apoptosis. Both of these functions help to reduce the possibility of tumorigenesis [4]. A study of 3281 tumors involving 12 tumor types found that the average mutation frequency of TP53 was about 42% [5]. A report from 10,225 patients with 32 different cancers from the Cancer Genome Atlas (TCGA) showed that TP53 mutations were more frequent in cancer patients with lower survival rates among all cancer types studied [3]. These data indicated the crucial role of the TP53 mutation in the occurrence and development of malignant tumor.

TP53 can be used for ultra-early screening of GC, and can be used to monitor postoperative recurrence of gastric cancer by monitoring free DNA mutations, and predicting the efficacy of paclitaxel combined with capecitabine in the treatment of advanced GC [6, 7]. Interestingly, some recent studies have shown that different immune responses are associated with TP53 mutation status [8–11]. Immunotherapy, as a new treatment for GC, has great potential in clinical application [12]. Although there are few studies on the relationship between TP53 mutation and the efficacy of immunotherapy in GC, there was increasing evidence suggested that the TP53 mutation can affect the immunophenotype of GC [13–15]. Although the mechanism of TP53 mutation affects the immunophenotypic regulation of GC remains unclear, considering the important role of TP53 in maintaining genomic stability, the change of immune genome expression pattern mediated by TP53 mutation may affect the immunophenotype of GC and lead to different clinical outcomes.

In our work, we paired the differential immune-related genes associated with TP53 mutation and studied the effect of this combination on the overall prognosis of GC. We developed a prognosis model contained 11 immune gene pairs based on TCGA datasets and used four independent cohorts from GEO database to validate its prognostic value for GC. This large sample, multicenter analysis may provide an important basis for the comprehensive management of GC.

## Materials and methods

### Data collection

In terms of the 375 GC tissues, their RNA-sequencing profile was obtained from The Cancer Genome Atlas (TCGA, https://portal.gdc.cancer.gov/). The complete prognostic information of 350 GC patients was available in the TCGA database. The TP53- alterated sample list was acquired from the cBioPortal (https://www.cbioportal.org/). The gene expression files and corresponding clinical data of four independent cohorts (GSE84437, n = 431; GSE62254, n = 300; GSE15459, n = 191; GSE26901, n = 100) were downloaded from the Gene Expression Omnibus (GEO) database (https:// www.ncbi.nlm.nih.gov/geo/). The downloaded profiles were all complied with the TCGA and GEO data access rules. The detailed clinical information of the above five datasets were displayed in Table 1. The data utilized in this work were obtained from public databases, so the approval from the local ethics committee was not needed.

**Table 1:** The clinical data of the 5 independent cohorts

	TCGA (n = 350)	GSE84437(n = 431)	GSE62254(n = 300)	GSE15459(n = 191)	GSE26901(n = 100)
Survival status					
Alive	204	224	148	96	45
Dead	146	207	152	95	55
Age					
> 65	189	150	97	108	23
< = 65	158	283	136	83	77
Gender					
Female	123	137	74	67	39
Male	224	296	159	124	61
Grade					
G1–2	134				
G3	207				
Stage T					
T1–2	90	49			
T3	161	92			
T4	95	292			
Stage N					
N0	103	80			
N1	93	188			
N2	72	132			
N3	71	33			
Stage M					
M0	312				
M1	23				
Stage TNM					
I–II	156		139	60	52
III	145		75	72	33
IV	35		19	59	15
Laurenclassificatio n					
Diffuse		102	122	75	11
Intestinal		119	105	98	74
Mixed		10	6	18	5
Perineural Invasion					
Yes			86		
No			147		
Lymphovascular					
Yes			171		
No			62		
Subtype					
Invasive				51	
Metabolic				40	
Proliferative				69	
Unstable				31	
Subgroup					
MP					39
EP					61
Adjuvant.chem					
Yes					37
No					63
Location					
Antrum					51
Body					34
Entire					4
Fundus					11

### Identification of differential expressed immune-related genes (DEIRGs)

The list of genes related to immune came from the ImmPort database (https://immport.niaid.nih.gov) (Additional file 1) and we extracted them from TCGA datasets. The “edgeR” R package was used to identifying DEIRGs in TP53-altered GC samples (n = 183) and without TP53-altered GC samples (n = 192). A false discovery rate (FDR) of < 0.05 was considered significant. The R package “cluster profile” was applied for DEIRGs annotation (Kyoto Encyclopedia of Genes and Genomes).

### Construction of immune gene pairs

We paired the DEIRGs. In each immune gene pair (IGP), if the former gene presented a higher expression relative to the latter one, the value was defined to 1. On the contrary, if the expression level of the former gene was lower compared to the latter one, the value was defined to 0. The IGPs with proportion of “0” or “1” less than 20% were excluded. Since the IGPs were generated by a pairwise comparison and were entirely based on the gene expression in the same patient, the gene expression profiles of different platforms did not need to be normalized.

### Construction and validation of the immune gene pairs prognostic model

The prognostic related immune gene pairs (PRIGPs) were identified by the univariate Cox regression analysis first [16]. P < 0.001 was considered to be significant. Next, the scope of PRIGPs was reduced by the least absolute shrinkage and selection operator (LASSO) algorithm, as well as penalty parameter tuning based on tenfold cross-validation [17]. Then we input the IGPs with nonzero regression coefficients to the multivariate cox regression analysis. After that, multivariate Cox regression coefficients were calculated to establish a risk score. Patients were divided into low-risk and high-risk groups based on the median risk score of the TCGA cohort (n = 350). LASSO regression analysis was carried out with the "glmnet" R package. R package “survminer” and “survivalROC” were employed to obtain the receiver operating characteristic (ROC) curve and the Kaplan–Meier survival curve. The prognostic model’s independent prognostic value was evaluated through the univariate and multivariate Cox regression analysis. The predictive capability and applicability of the prognostic model were verified based on four independent external validation cohorts including GSE84437 (n = 431), GSE62254 (n = 300), GSE15459 (n = 191) and GSE26901(n = 100).

### Immune infiltration analysis between different risk groups

The CIBERSORT algorithm was used for quantifying 22 kinds of immune cell infiltration proportions of all the included 1372 GC samples (TCGA, n = 350; GSE84437, n = 431; GSE62254, n = 300; GSE15459, n = 191; GSE26901, n = 100) [18–20]. We used p < 0.05 as the threshold to judge the accuracy of prediction of immune cell infiltration, the samples with p < 0.05 could be used for subsequent analysis.

## Results

### Identification of DEIRGs associated with TP53

**Fig. 1:**
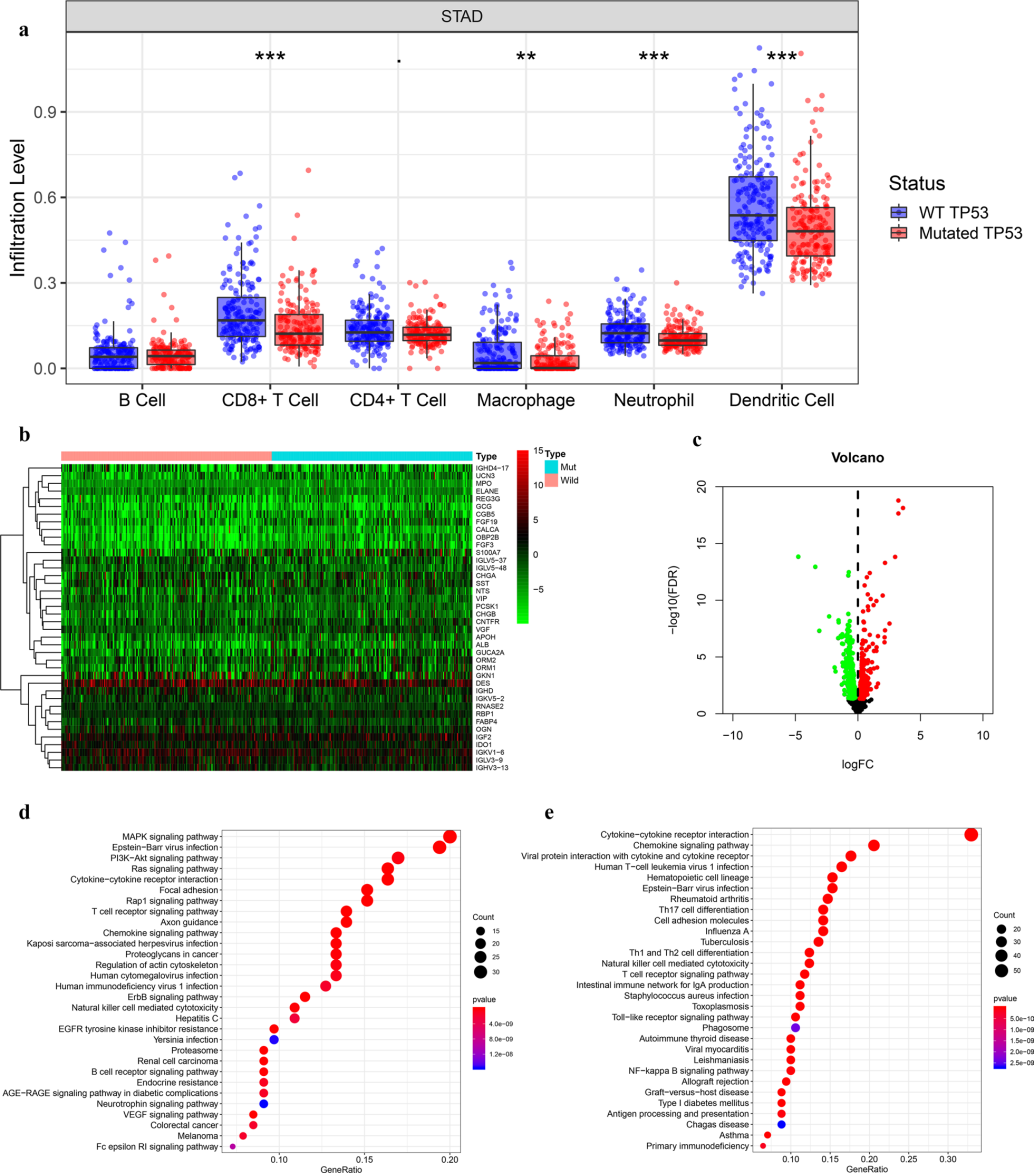
Identification of differential expressed immune-related genes (DEIRGs) **a** The boxplot of immune cell infiltration in with and without TP53 mutation GC tissues (The image downloaded from TIMER, https://cistrome.shinyapps.io/timer/) **b**, **c** The heatmap and vol plot of DEIRGs. **d** The bubble plot of KEGG pathways for the DEIRGs up-regulated in TP53-mutation GC. **e** The bubble plot of KEGG pathways for the DEIRGs up-regulated in without TP53-mutation GC

**Fig. 2:**
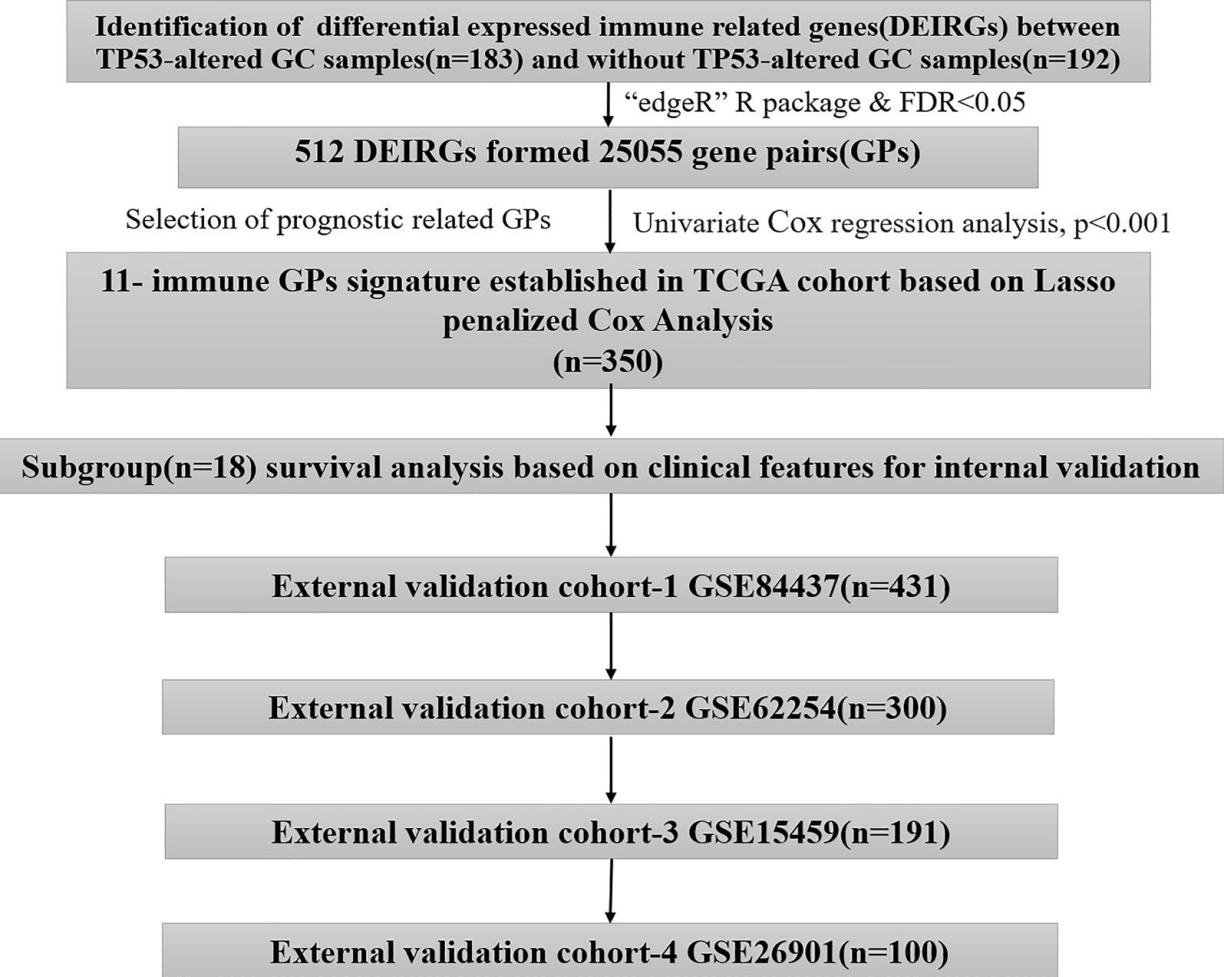
The workflow chart of this study

Tumor IMmune Estimation Resource (TIMER) algorithm could predict the composition of infiltrating immune cells in each tumor sample based on the gene expression profile data of tumor samples [21, 22]. We used the TIMER algorithm to estimate the abundances of six immune infiltrates (B cells, CD4 + T cells, CD8 + T cells, Neutrophils, Macrophages, and Dendritic cells) in GC samples, and found that the infiltration level of CD8 T cells, dendritic cells, neutrophils and Macrophages exhibited obviously difference in the mutated-TP53 and WT-TP53 GC tissues (Fig. 1a). Next, we identified 512 DEIRGs in TP53-altered and without TP53-altered GC samples. Among them, 216 genes were up-regulated in 183 TP53-altered GC samples, and 296 genes were up-regulated in 192 without TP53-altered GC samples (Fig. 1b, c). The KEGG pathway enriched by up-regulated immune genes in GC samples with TP53 mutation and without TP53 mutation was also different (Fig. 1d, e). These results confirmed the hypothesis of the immunophenotype of GC may affected by the TP53 mutation. The workflow of our paper as shown in Fig. 2.

### Construction of the immune gene pairs prognostic model in TCGA cohort

**Fig. 3:**
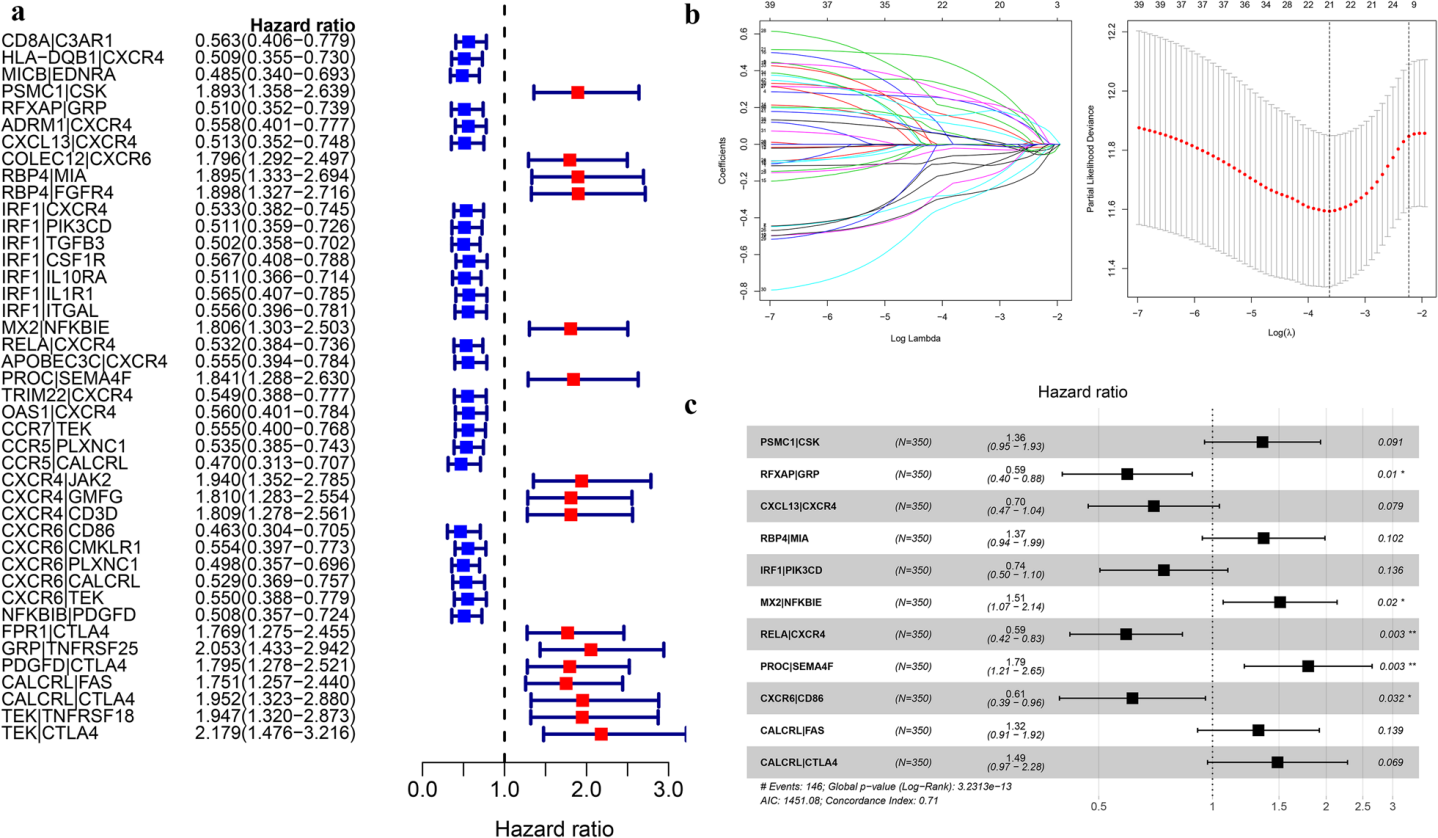
The building process of the 11 immune gene pairs prognostic signature (**a**). The forrest plot of the univariate Cox analysis. **b** Lasso regression analysis. **c** Multivariate Cox regression analysis

After a pairwise comparison, 512 DEIRGs formed 25,055 gene pairs (GPs), 105,761 GPs were excluded from 130.816 GPs because the proportion of "0" or "1" was less than 20%. The univariate Cox regression analysis revealed the significant correlation of 42 GPs with overall survival (p < 0.001) (Fig. 3a). The 42 GPs were further reduced by Lasso penalty Cox regression analysis, among which 21 GPs were repeated more than 900times after 1000 times tenfold cross-validation (Fig. 3b). Finally, a risk score was constructed after the selection of 11 GPs by step-by-step multivariate Cox regression (Fig. 3c). The median risk score-0.948 was used to divide the GC patients into two groups, low-risk and high-risk. The specific calculation formula of risk score as shown in Table 2.

**Table 2:** The list of gene pairs and corresponding coefficient

Gene pairs	Coef
PSMC1|CSK	0.304948
RFXAP|GRP	– 0.51969
CXCL13|CXCR4	– 0.35784
RBP4|MIA	0.311735
IRF1|PIK3CD	– 0.29637
MX2|NFKBIE	0.412191
RELA|CXCR4	– 0.52646
PROC|SEMA4F	0.583495
CXCR6|CD86	– 0.48794
CALCRL|FAS	0.280125
CALCRL|CTLA4	0.396375

### Prognostic assessment of the model in the TCGA cohort

**Fig. 4:**
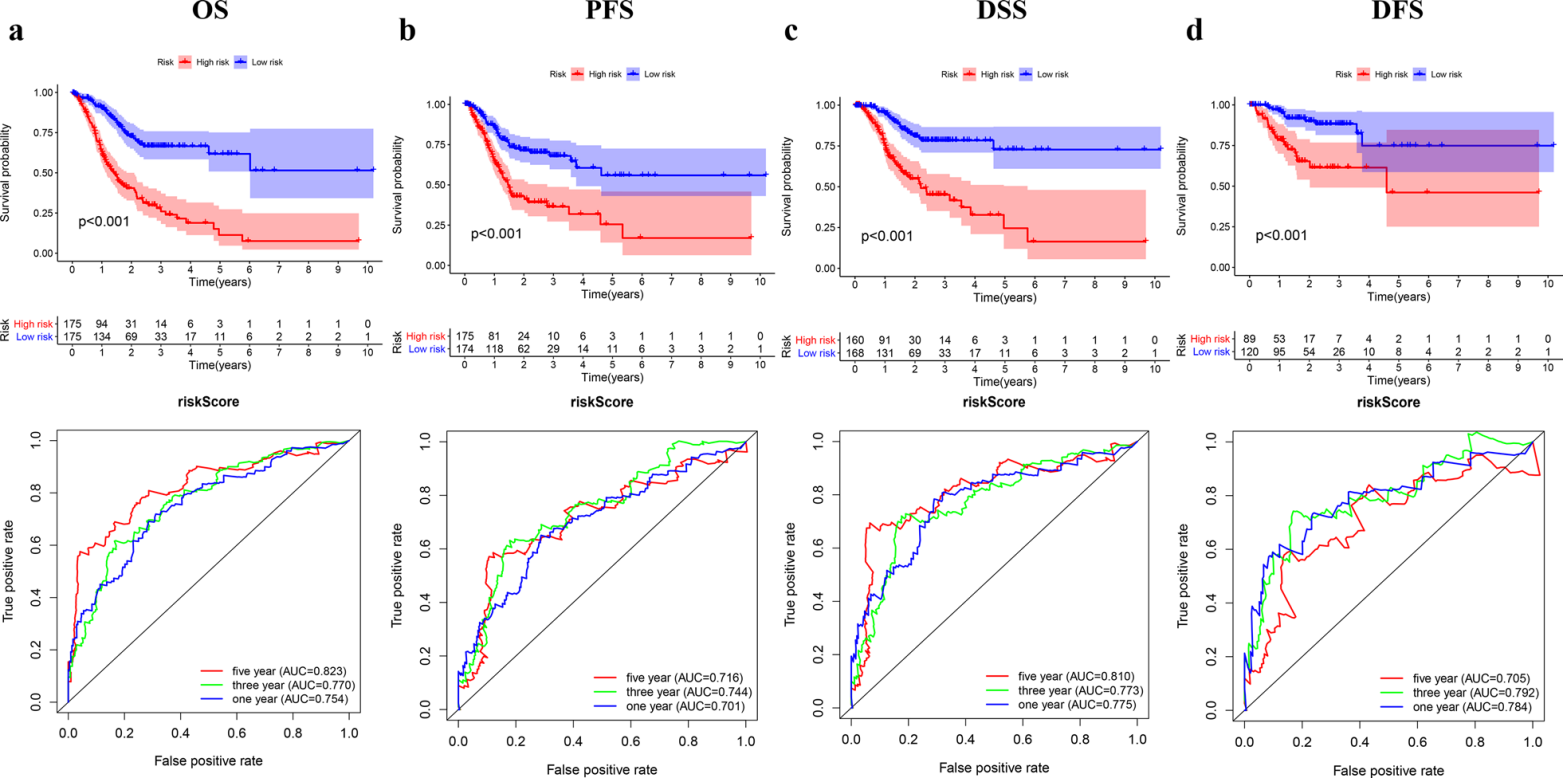
The prognostic assessment of the signature in the TCGA cohort. **a**The Kaplan–Meier survival analysis and time-dependent ROC analysis of the signature for predicting the OS of patients in the TCGA cohort. **b **The Kaplan–Meier survival analysis and time-dependent ROC analysis of the signature for predicting the PFS of patients in the TCGA cohort. **c** The Kaplan–Meier survival analysis and time-dependent ROC analysis of the signature for predicting the DSS of patients in the TCGA cohort. **d** The Kaplan–Meier survival analysis and time-dependent ROC analysis of the signature for predicting the DFS of patients in the TCGA cohort

Four prognostic indicators (PFS, progression-free survival; DFS, disease-free survival; DSS, disease special survival; OS, overall survival) were used to evaluate the prognostic value of the model for GC. As shown in Fig. 4, the high-risk group obtained lower values of OS, DSS, DFS, and PFS than the low-risk group (Fig. 4a–d). The area under curve (AUC) values for the model predicting OS at 1, 3 and 5 years were 0.754, 0.770 and 0.823 respectively. The AUC values for the model predicting PFS at 1,3 and 5 years were 0.701,0.744 and 0.716 respectively. The model predicting DSS obtained a result of 0.775,0.773 and 0.810 correspondingly. The model predicting DFS got a result of 0.784,0.792 and 0.705 (Fig. 4a–d). These results demonstrated the good performance of this prognostic model.

### Internal validation of the prognostic model in the TCGA cohort

**Fig. 5:**
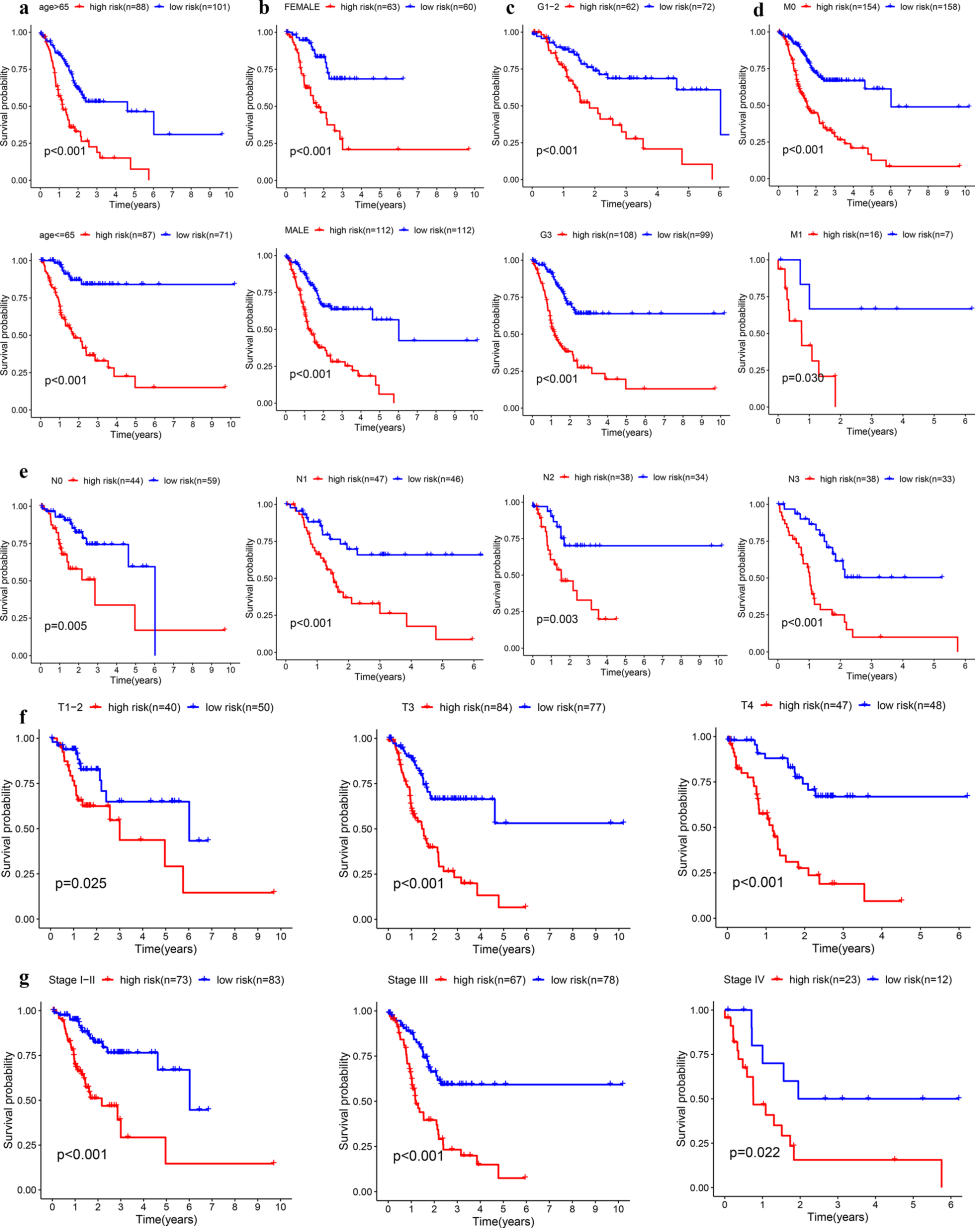
Subgroup survival analysis based on clinical features for internal validation. **a** Age, **b** Gender, **c** Grade, **d** stage M, **e** stage N, **f** stage T, **g** stage TNM

**Fig. 6:**
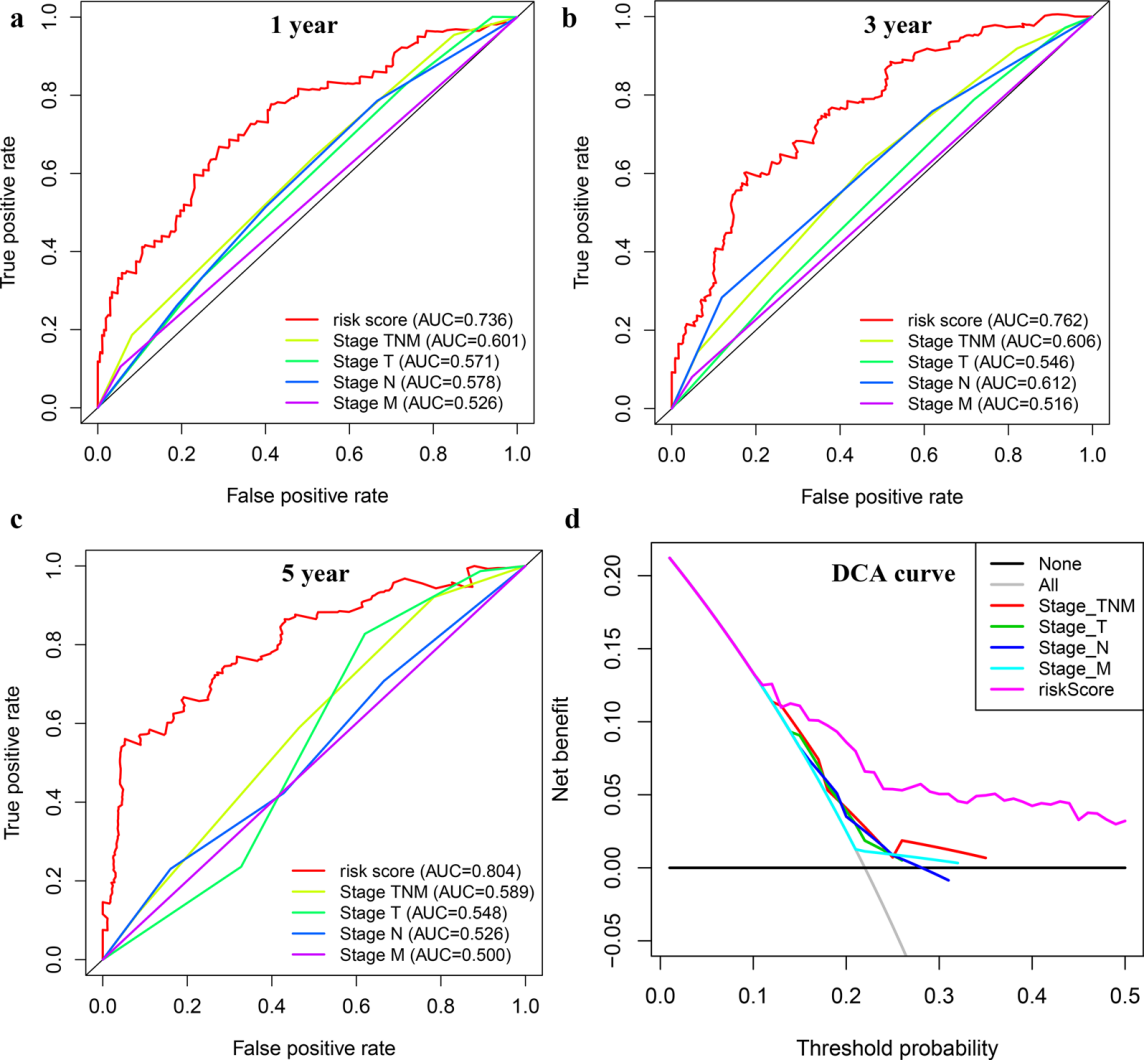
Comparison of the prognostic model and TNM stage system. **a** 1-year time-dependent ROC curve, **b** 3-year time-dependent ROC curve, **c** 5-year time-dependent ROC curve, **d** DCA curve

The clinical features were used to divide TCGA-GC patients into 18 subgroups. High-risk patients had significantly lower OS than low-risk patients in each subgroup (Fig. 5a–g). By observing the time-dependent ROC curves, we found that the AUC value of risk score was the highest in predicting OS of patients with GC at 1, 3 and 5 years, which showed that the prediction accuracy of risk score is higher than the existing TNM staging prognosis evaluation system (Fig. 6a–c). The Decision Curve Analysis (DCA) [23] were also confirmed that the risk score could bring the greatest clinical net benefit to patients (Fig. 6d). The general applicability of the prognostic model was preliminarily confirmed.

### External validation of the prognostic model in four independent cohorts

**Fig. 7:**
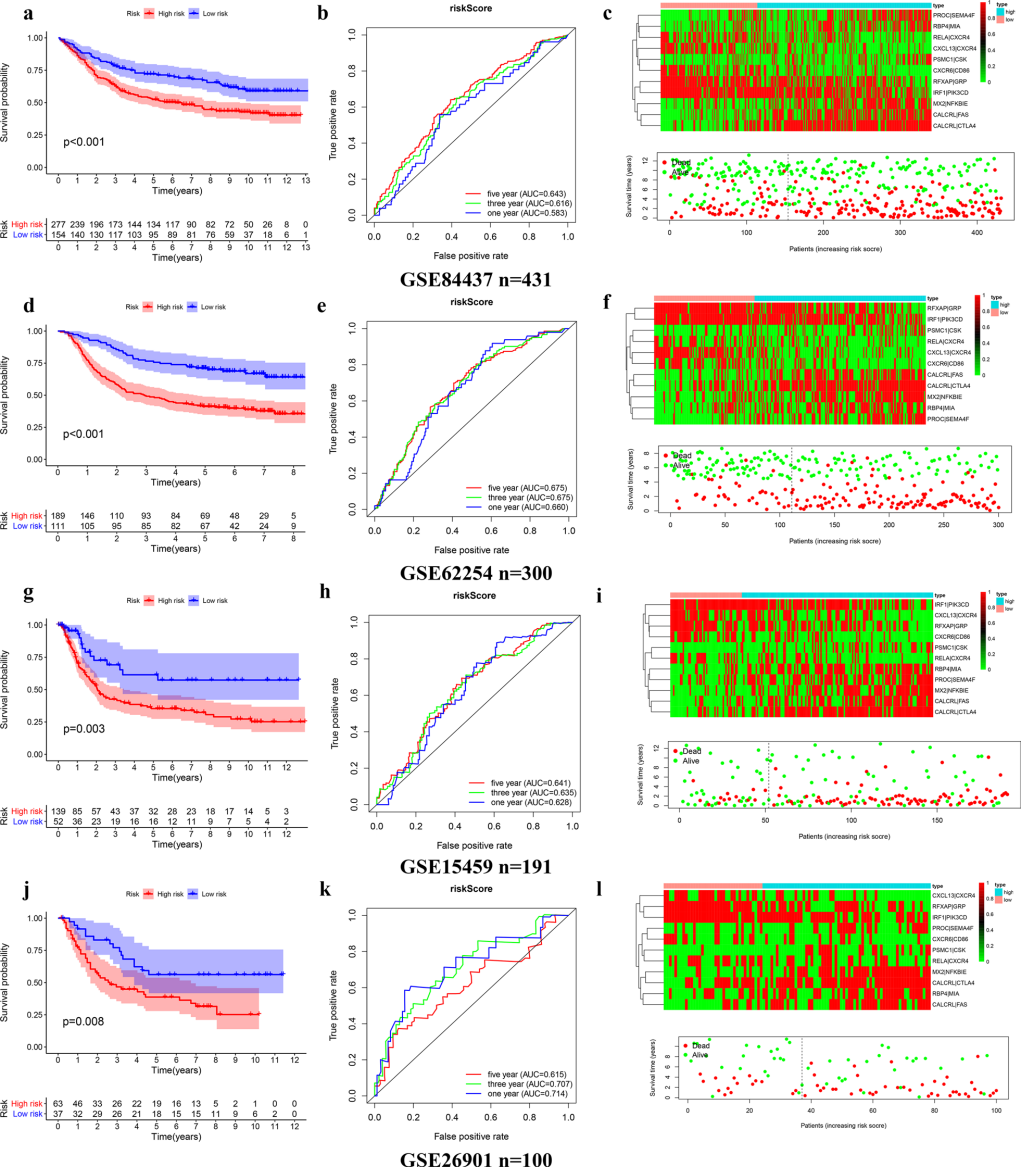
The Kaplan–Meier survival analysis, time-dependent ROC analysis, and the heatmap, survival status of patients in four independent external validation cohorts, **a**–**c** GSE84437 cohort, **d**–**f** GSE62254 cohort, **g**–**i** GSE15459 cohort, **j**–**l** GSE26901 cohort

**Fig. 8:**
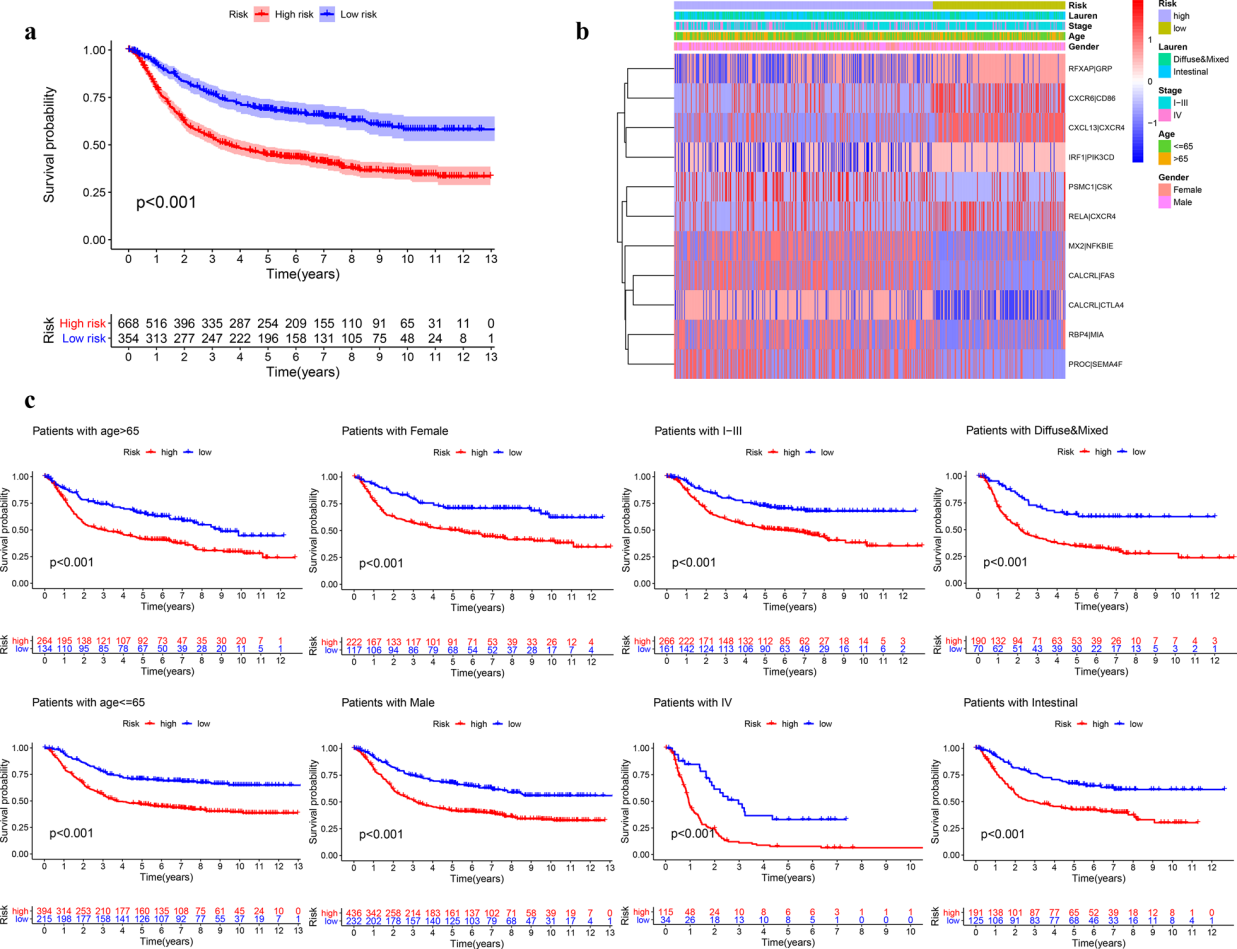
The Prognostic assessment of the signature in the entire-external validation samples. **a** The Kaplan–Meier survival analysis, **b** Heatmap, **c** clinical grouping validation

The risk score of GC patients in each independent cohort was calculated. These patients were classified into two groups, high-risk and low-risk, on the basis of the unified cutoff-0.948. All high-risk patients had significantly lower OS values than low-risk patients in each independent cohort (Fig. 7a, d, g, j). In the GSE84437 cohort (n = 431), the area under curve (AUC) values for the model predicting OS at 1, 3 and 5 years were 0.583,0.616 and 0.643 respectively (Fig. 7b). In the GSE62254 cohort (n = 300), the results were 0.660, 0.675 and 0.675 correspondingly (Fig. 7e). The results of the GSE15459 cohort (n = 191) were 0.628,0.635 and 0.641 (Fig. 7h). The results of the GSE26901 cohort (n = 100) were 0.714,0.707 and 0.615 (Fig. 7k). The risk of death increased with the increase of risk score (Fig. 7c, f, i, l). We pooled four independent validation cohort (a total of 1022 patients) for analysis, and found that the survival difference between high-risk group and low-risk group was still significant (Fig. 8a, b). The OS of high-risk patients in each subgroup divided by clinical traits were also significantly lower than that in the low-risk group (Fig. 8c). These results further confirmed the stability and general applicability of the our established prognostic model.

### The prognostic model as an independent prognostic indicator

**Fig. 9:**
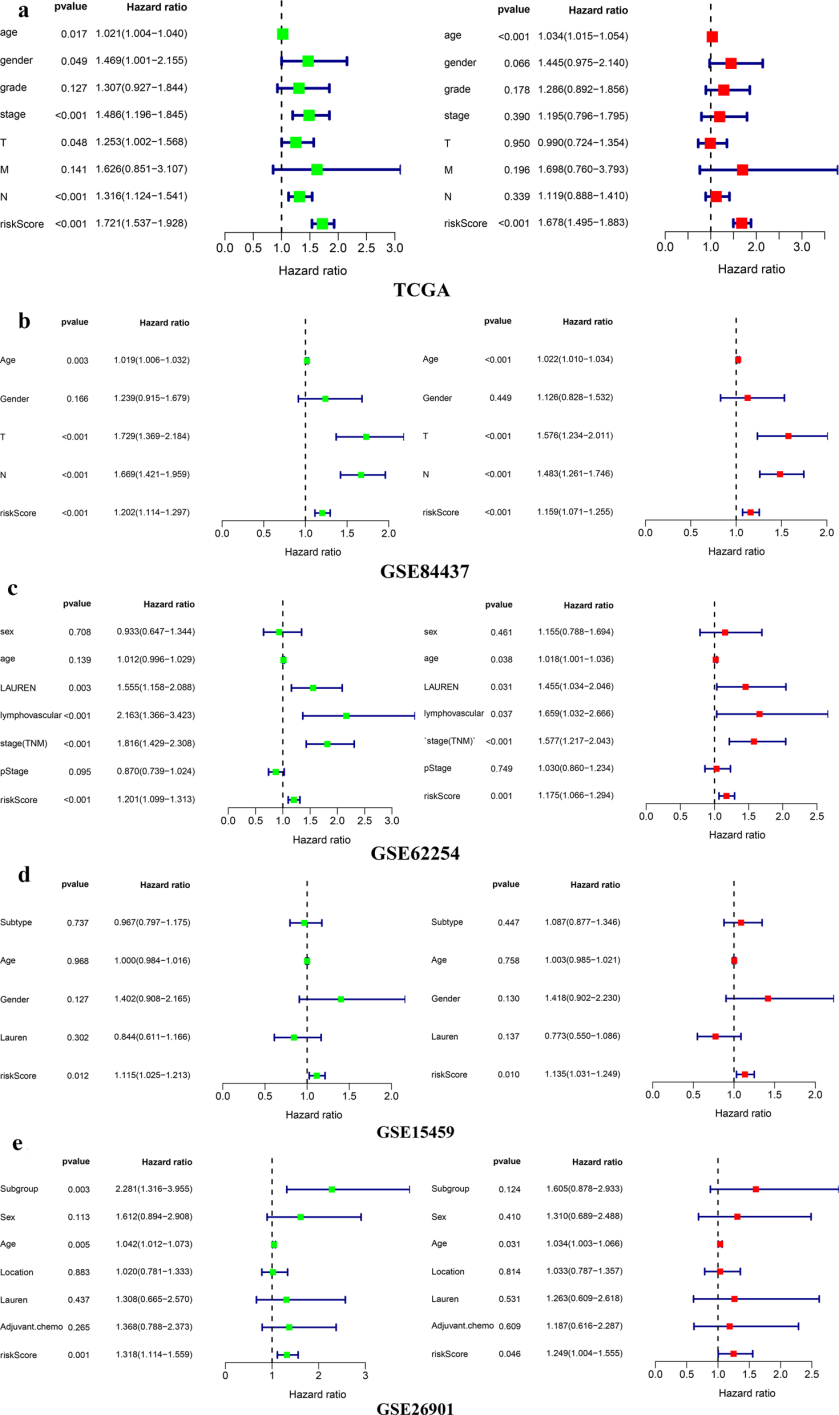
Independence validation of the prognostic signature in the 5 independent cohort. **a** The forrest plot of the univariate and multivariate Cox analysis in TCGA. **b** The forrest plot of the univariate and multivariate Cox analysis in GSE84437. **c** The forrest plot of the univariate and multivariate Cox analysis in GSE62254. **d** The forrest plot of the univariate and multivariate Cox analysis in GSE15459. **e** The forrest plot of the univariate and multivariate Cox analysis in GSE26901. *Green represents univariate analysis, and red represents multivariate analysis

We included the risk score and other clinical factors into the univariate and multivariate Cox regression analysis, as the results suggested that the risk score was an independent prognostic indicator in the TCGA, GSE84437, and GSE62254 cohort (Fig. 9a–c). However, in the GSE15459 and GSE26901 cohorts, the risk score could be considered as an independent prognostic risk factor for GC only when TNM staging was excluded from multivariate Cox regression analysis (Fig. 9d, e).

### The difference of immune cell infiltration between two groups

**Fig. 10:**
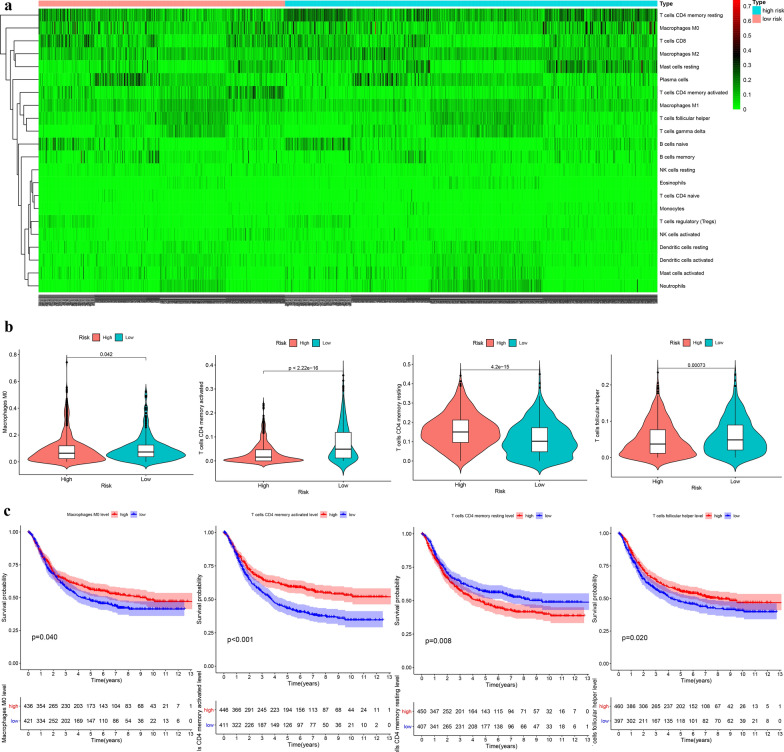
The difference of immune cell infiltration between high- and low-risk groups. **a** The heatmap of immune infiltration landscape. **b** The vioplot of the difference of the infiltration level of the T cells memory activated, T cells memory resting, T cells follicular helper, and Macrophage M0 between high- and low-risk groups. **c** The Kaplan–Meier survival analysis of the infiltration level of the T cells memory activated, T cells memory resting, T cells follicular helper, and Macrophage M0

With p < 0.05 as the threshold, 335 samples with p value greater than 0.05 as predictive inaccurate samples were excluded. We integrated the immune infiltration of the remaining 413 low-risk GC tissues and 624 high-risk GC tissues (Fig. 10a). The high-risk GC tissues exhibited a lower infiltration level of T cells follicular helper, T cells memory activated, and Macrophage M0, but a higher infiltration level of T cells memory resting than the low-risk GC tissues (Fig. 10b). Coincidentally, it was found that the higher infiltration level of T cells memory resting was not conducive to the prognosis of GC patients, while the higher infiltration level of Macrophage M0, T cells memory activated, and T cells follicular helper showed an opposite result (Fig. 10c). Therefore, the different clinical outcomes of GC patients may be related to tumor immunity.

### Clinical correlation analysis for the risk score

**Fig. 11:**
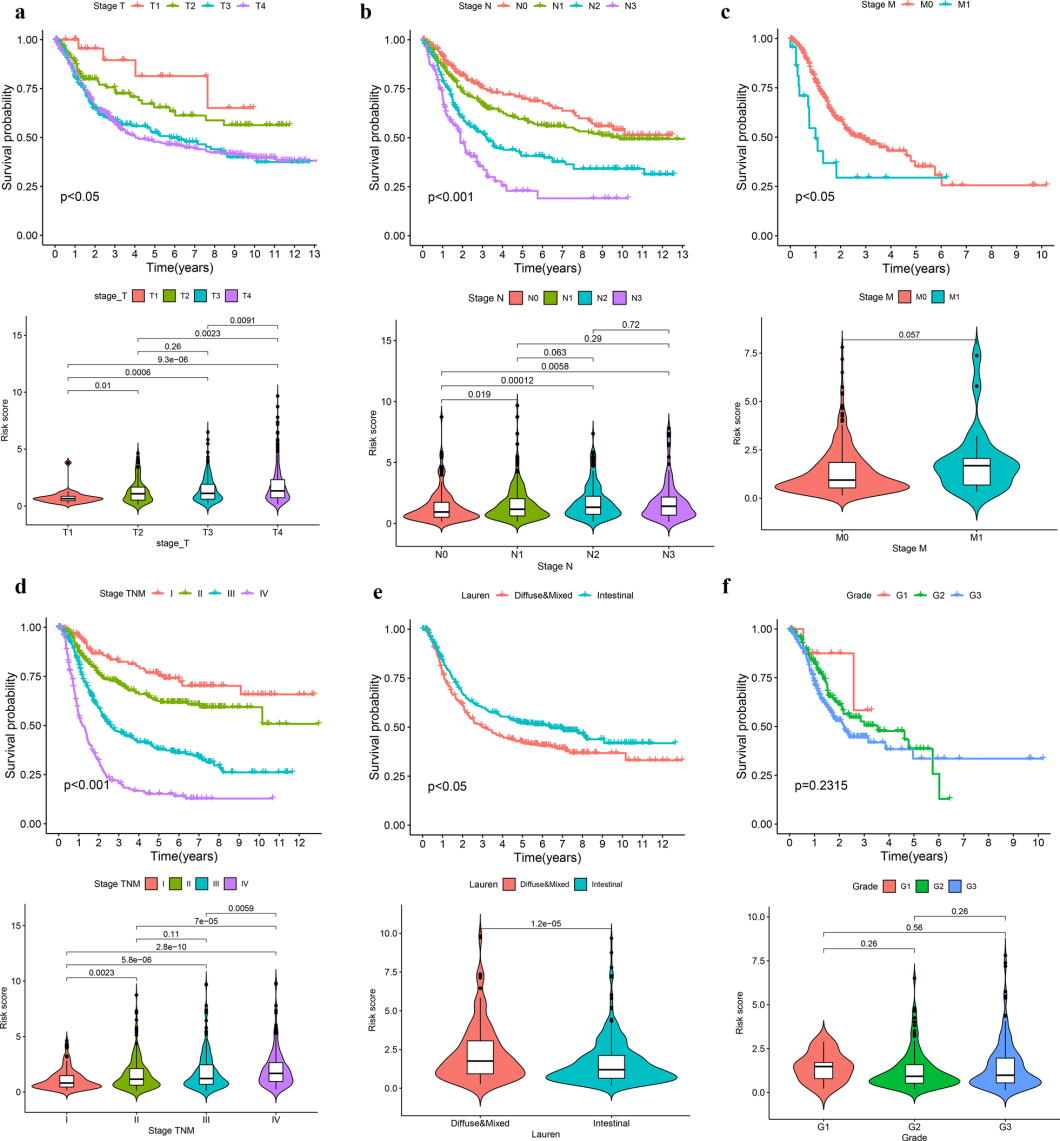
Clinical correlation analysis. **a** Stage T. **b** Stage N. **c** Stage M. **d** Stage TNM. **e** Lauren classification. **f** Grade

The stage T, stage N, stage M, and stage TNM were closely related to the prognosis of GC patients, and the patients’s risk score were positively correlated with the stage T, stage N, stage M, and stage TNM (Fig. 11a–e). The patients with lauren classification’s diffuse and mixed type had poorer prognosis and higher risk score. However, there was no significant correlation between tumor grade and prognosis (Fig. 11f).

### The prognostic signature’s underlying molecular mechanism

**Fig. 12:**
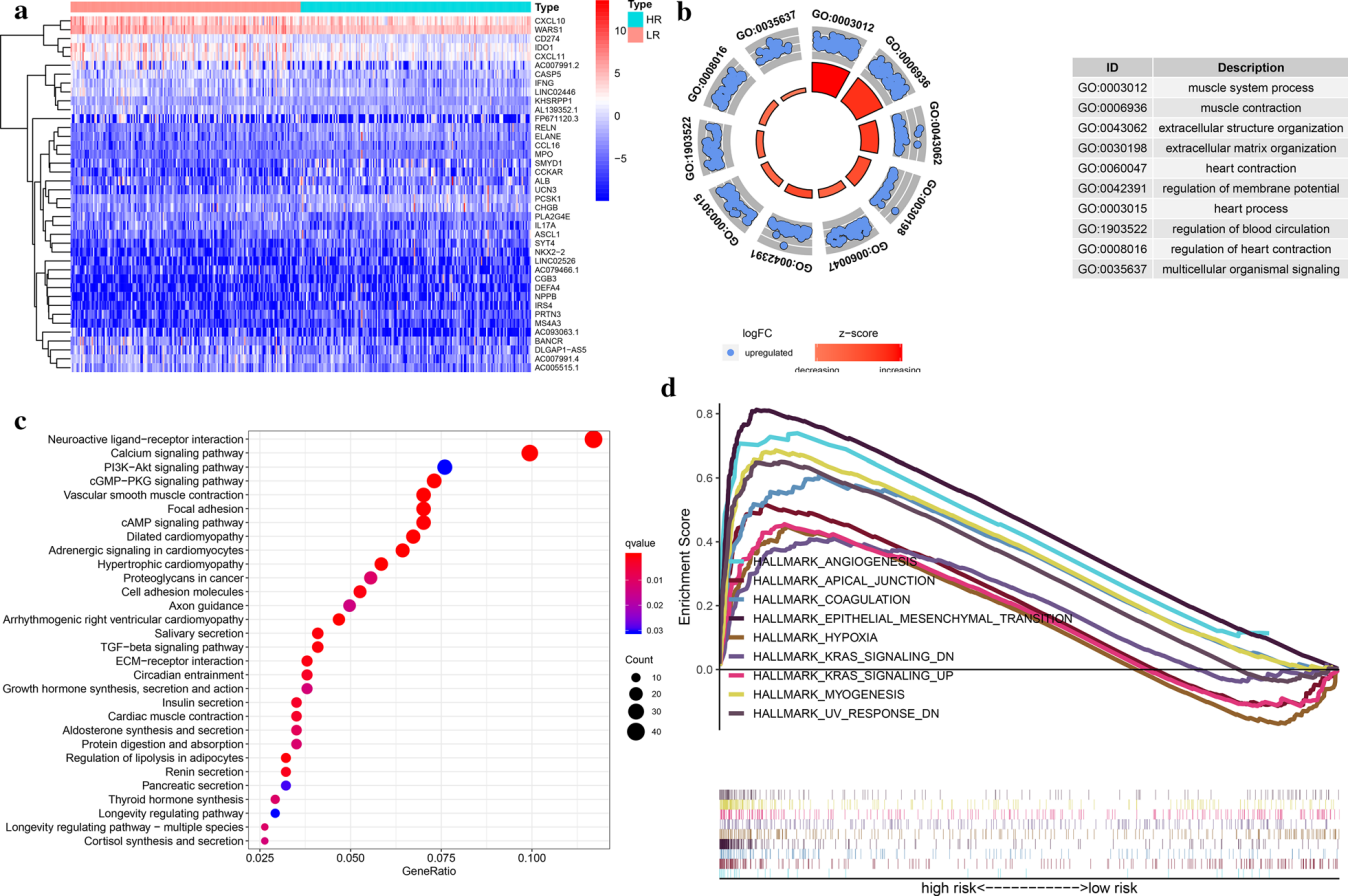
The prognostic model’s underlying molecular mechanism. **a** The heatmap of DEGs between high- and low-risk groups. **b** The GO annotation of the DEGs up-regulated in the high-risk group. **c** The KEGG pathway enrichment analysis for the DEGs up-regulated in the high-risk group. **d** The 9 gene sets identified by GSEA positively correlated with the high-risk group

We identified the differential expressed genes (DEGs) in different risk groups using the R package “limma”(fdrFilter = 0.05, logFCfilter = 1) (Fig. 12a). Among 996 DEGs, 929 genes were up-regulated in high-risk group and 67 genes were down-regulated (Additional file 2). We conducted Gene Ontology (GO) annotation and KEGG pathway enrichment analysis for the DEGs up-regulated in the high-risk group with the R package “clusterProfiler”, and found that the GO terms related to extracellular structure organization and extracellular matrix organization were associated with high-risk group (Fig. 12b). The activity of calcium signaling pathway, cGMP-PKG signaling pathway, and PI3K-Akt signaling pathway were enhanced in the high-risk group (Fig. 12c). Besides, we also identified 9 gene sets positively correlated with the high-risk group by gene sets enrichment analysis (GSEA), such as the hallmark of angiogenesis, hypoxia, and epithelial-mesenchymal transition (EMT), etc.(Fig. 12d; Table 3), the “h.all.v7.1.symbols.gmt” were regarded as an reference.

**Table 3:** The 9 gene sets identified by GSEA showed positive correlation with the high-risk group

Name	ES	NES	NOM p-val	FDR q-val	FWER p-val
HALLMARK_MYOGENESIS	0.685	2.302	0	0	0
HALLMARK_EPITHELIAL_MESENCHYMAL_TRANSITION	0.812	2.261	0	0.001	0.005
HALLMARK_ANGIOGENESIS	0.739	2.017	0	0.008	0.032
HALLMARK_COAGULATION	0.608	1.973	0	0.0103	0.048
HALLMARK_KRAS_SIGNALING_DN	0.412	1.652	0.004	0.081	0.276
HALLMARK_UV_RESPONSE_DN	0.650	2.042	0.004	0.008	0.025
HALLMARK_APICAL_JUNCTION	0.515	1.815	0.017	0.035	0.146
HALLMARK_KRAS_SIGNALING_UP	0.455	1.609	0.037	0.091	0.323
HALLMARK_HYPOXIA	0.448	1.545	0.046	0.098	0.399

## Discussion

Gastric cancer (GC) is a typically malignant tumor of the digestive tract, since most GC patients were diagnosed in the advanced stage with a poor prognosis [24]. In recent years, comprehensive genomic research of GC has received growing attention [25], but the valuable biomarkers can be assisted in clinical diagnosis and treatment were still lacking [26]. The current methods of predicting prognosis cannot fully reflect the heterogeneity of GC, which usually difficult to accurately evaluate the clinical outcomes of GC [27]. Finding an precisely prognostic evaluation system could optimize the use of medical resources, which has important scientific and clinical significance [28].

Growing evidences have been suggested that the TP53 mutation can affect the immunophenotype of GC [13–15], but its mechanism still unclear. Maintaining genomic stability is one of the most important function of TP53. In the beginning of our study, we posed a hypothesis that the change of immune genome expression pattern mediated by TP53 mutation may affect the immunophenotype of GC and lead to different clinical outcomes. As expected, we found different infiltration levels of several immune cells in the GC tissues with and without TP53 mutation, as well as the expression levels of immune-related genes. There are few reports about the effect of this difference on the prognosis of GC so far. Next, we focused on the construction and validation of the prognosis model according to this clue, for improving the currently used prognosis evaluation system.

Considering the units of gene expression were not standardized, the traditional prognostic signature based on gene expression levels with limited applicability for the assessment of the prognosis of GC [29]. Developing a new prognostic model with universal applicability has become an urgent need in the research field of GC. Since the gene pairs were generated by a pairwise comparison based on the gene expression in the same patient, the gene expression profiles of different platforms did not need to be normalized [30], which made this method more convenient to use.

A total of 11 immune gene pairs were screened out to construct a risk score through multivariate Cox regression analysis, Lasso regression and univariate Cox analysis. In the TCGA cohort, the prognostic model showed good predictive performance for OS, PFS, DSS, and DFS of GC patients. To further evaluate the models’ applicability, we carried out subgroup analysis and external validation. The results of subgroup analysis showed that the model was suitable for GC patients with different characteristics. It is worth mentioning that our model could capable of distinguish between GC patients with good prognosis and poor prognosis in all four independent external validation cohort. Besides, the risk score was identified to be an independent prognostic indicator in each independent cohort. These evidences indicated that the prognostic model has great potential for clinical application. Another important finding is the two groups’ significant difference in the proportion of immune cell infiltration, which could be explained by the prognosis of GC patients. This finding demonstrated that we could evaluate the immune response of GC tissue according to the prognostic model, which has important guiding significance for the development of individualized treatment plan for GC patients. We also compared it with the TP53 associated 9 immune gene signature constructed by Nie et al. [29]. The AUC values for Nie’s TP53 associated 9 immune gene signature predicting OS at 1,3 and 5 years were 0.691,0.704 and 0.742 respectively, while the AUC values for our model predicting OS at 1,3 and 5 years were0.754,0.770 and 0.823 respectively, which confirmed the superiority of the model further.

In order to reveal the potential reasons for the difference of prognosis between high-risk group and low-risk group, we observed the distribution of risk score in GC patients with different clinical characteristics. Although there was no significant correlation between risk score and tumor differentiation, risk score was positively correlated with the stage T, stage N, stage M, and stage TNM, and the risk score of patients with lauren classification’s diffuse and mixed type were also significantly higher than patients with lauren classification’s intestinal type. These results suggested that we can predict the degree of tumor invasion, lymph node metastasis and pathological classification according to the risk score. GO annotation showed that the terms related to extracellular structure organization and extracellular matrix organization were up-regulated in the high-risk group, which indicated that the polarity and skeleton structure of GC cells in high-risk group are more likely to change, thus showing high invasiveness [31]. KEGG pathway enrichment analysis demonstrated that the risk score may stimulated the proliferation of GC cells by activating the PI3K-Akt signaling pathway [32], which consistent with the presentation of GSEA’s results suggested that the expression of angiogenesis and hypoxia related gene sets were up-regulated in the high-risk group. Because the continuous proliferation of tumor cells needs angiogenesis to provide nutrients, in the process of GC tumor proliferation, the increase of the distance between tumor cells and the surrounding stromal vessels will lead to hypoxia in the tumor [33]. In addition, the EMT related gene sets were also up-regulated in the high-risk group, indicated that the patients with higher risk score were prone to invasion and metastasis [34].

A few of genes in the model have been confirmed to play a role in the occurrence and progression of GC. For example, Izumi [35] reported that the stimulation of CXCR4 promoted the invasive ability of GC cells. Liu [36] found that the PIK3CD was a core oncogene involved in the progression of GC. Yamaguchi [37] demonstrated that the macrophages M2 (CD86( +)) could contribute to the proliferation and progression of GC. Wang [38] demonstrated the correlation of the downregulation of FAS expression with increased lymph node and distant metastases of GC as well as less histological differentiation and gender (male). Wei [39] demonstrated the correlations of the high CXCL13 expression with lower OS and larger tumor diameter in GC.Yuan [40] supported the role of IRF-1 in hindering GC metastasis, which was achieved through the reduction of Wnt/β-catenin signaling and the downregulation of MIR17HG-miR-18a/miR-19a axis expression. Jin [41] identified CXCR6 to be an independent prognostic factor for poor survival in GC patients, which may played a role in advancing GC metastasis by means of epithelial-mesenchymal transition. Xiang [42] demonstrated that the activation of CXCR4 could promote the metastasis of GC. As there are few studies on the remaining moleculars in GC and even cancers, their specific biological effects are still unclear. However, with the gradual deepening of the understanding of these new biomarkers, it will effectively promote the development of individualized and precise treatment for GC.

To sum up, this study was a retrospective study with large sample and multi centers. A total of 1372 GC patients from 5 independent cohort included in our work. We constructed and validated a valuable system to evaluate the prognosis of GC. Considering the stability and general applicability of the model, it may become a widely used tool in clinical practice. However, there are still some deficiencies in this study. First, although the overall effect of external validation in GEO database was satisfactory, the accuracy of the model in GEO external validation cohorts was not as good as TCGA cohort. Second, the gene pairs were generated by a pairwise comparison had important prognostic value for GC found by our work, but its underlying mechanism was not fully revealed by our paper, which needs further study. Third, our study was still a retrospective study and cannot replace a prospective multicenter clinical trials.

## Conclusion

Our study proposed a novel system for evaluating the prognosis of gastric cancer. Considering its stability and general applicability, which may become a widely used tool in clinical practice.

## Supplementary Information

**Additional file 1**: The immune related gene list obtained from the ImmPort database.

**Additional file 2**: The differential expressed genes in different risk groups.

**Additional file 3**: The timedependent ROC curve for the signature predicting OS of meta-GEO cohort (1022 patients).

**Additional file 4**: Source code (https://github.com/huojunyu/JTRM-D-21-00055).

## Data Availability

The datasets analysed for this study were obtained from The Cancer Genome Atlas (TCGA)(https://portal.gdc.cancer.gov/), the cBioPortal (https://www.cbioportal.org/) and Gene Expression Omnibus (GEO) (https://www.ncbi.nlm.nih.gov/geo/).

## Abbreviations

GC: Gastric cancer
TCGA: The Cancer Genome Atlas
GEO: Gene Expression Ominibus
DEIRGs: Differential expressed immune-related genes
DEGs: Differential expressed genes
KEGG: Kyoto Encyclopedia of Genes and Genomes
GO: Gene Ontology
GSEA: Gene Sets Enrichment Analysis
EMT: Epithelial-mesenchymal transition
TNM: Tumor Lymph Node Metastasis
TIMER: Tumor IMmune Estimation Resource
GPs: Gene pairs
IGP: Immune gene pair
PRIGPs: Prognostic related immune gene pairs
ROC: Receiver operating characteristic
AUC: Area under curve
OS: Overall survival
DSS: Disease special survival
DFS: Disease free survival
PFS: Progression free survival
RFS: Recurrent free survival
