# Supplementary figures and images for: Eleven immune-gene pairs signature associated with TP53 predicting the overall survival of gastric cancer: a retrospective analysis of large sample and multicenter from public database
Source: J Transl Med. 2021 Apr 29;19:183. doi: 10.1186/s12967-021-02846-x (PMC8086088; doi:10.1186/s12967-021-02846-x)

## riskScore

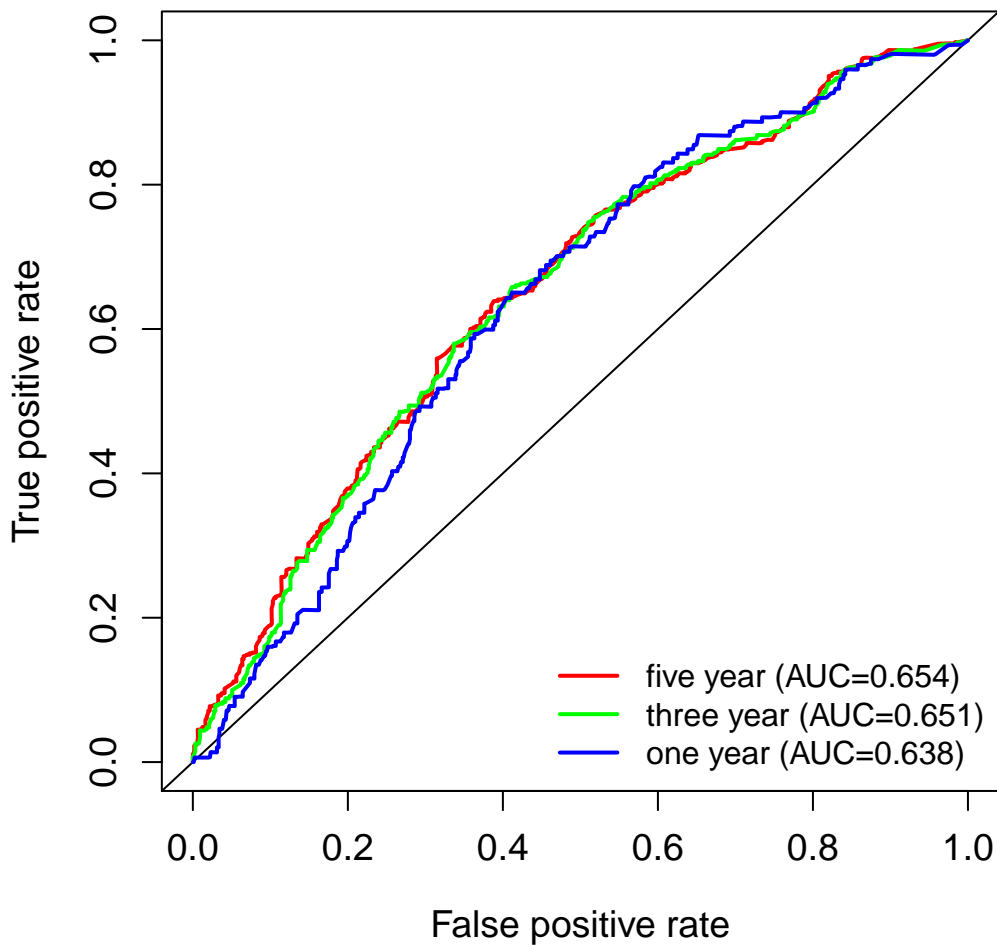

Supplement: Supplementary file 3 — Additional file 3: The timedependent ROC curve for the signature predicting OS of meta-GEO cohort (1022 patients). [file 12967_2021_2846_MOESM3_ESM.pdf]
